# Factors affecting oral examinations and dental treatments among older adults in Israel

**DOI:** 10.1186/s13584-019-0312-x

**Published:** 2019-05-10

**Authors:** Shosh Shahrabani

**Affiliations:** The Economics and Management Department and Head of Research Authority, The Max Stern Yezreel Valley College, P.O. 19300, Emek Yezreel, Israel

**Keywords:** Oral health, Older adults, Israel

## Abstract

**Background:**

Routine dental examinations are important for early diagnosis and treatment of dental problems among older adults in order to improve their quality of life and avoid costly future treatments. In Israel, a significant percentage of adults do not seek dental care.

**Methods:**

The study is based on a 2017 telephone survey conducted among people aged 50–75 from different population groups in Israel to examine their health beliefs and attitudes toward dental treatments.

**Results:**

The results show that among older adults the decision to undergo routine dental checkups is affected by socio-demographic status and health beliefs regarding dental health. Lower age, higher income levels, and Jewish religion predicted higher chances of frequent checkups. In addition, those who saw the benefits of routine checkups, believed that dentists were readily available, and had higher levels of health motivation were more likely to seek out routine dental care.

**Conclusions:**

According to the recent dental reform in Israel, people aged 75 and over are eligible for subsidized dental treatments. To enhance the frequency of dental checkups among older adults, it is recommended to provide this subsidized coverage for adults under age 75. In addition, planning dental health services for individuals in this age group should be based upon their accepted beliefs and values. Moreover, systematic health education through the media and health maintenance organizations should specifically target this population group to encourage them to undergo dental checkups more frequently.

## Background

Routine oral examinations and dental treatment for the older adult population play an important role in preventing health issues, improving quality of life, and reducing healthcare system costs [1, 2]. The demographic revolution reflected in the growing numbers of older adults in the general population poses new challenges for dental health professionals regarding how to provide appropriate and affordable oral care [3]. Poor oral hygiene can lead to overall deficient health and enhance the risk of chronic illness, mostly due to common risk factors such as infections [2, 4].

International comparisons provided by the WHO indicate that Israel has a high rate of dentists per capita compared to countries with similar welfare status.^1^ Yet, oral hygiene among older adults in Israel is poor. About one-third of people aged 65 and over reported difficulties in chewing food [5]. According to the Israel Ministry of Health’s 2013 report, costs for dental medicine constitute around 26% of the family healthcare basket. Currently, the level of expenditure for private healthcare, including dental treatment, among low and high income individuals is similar [6]. Only a small percentage of older adults in need of dental services are entitled to dental treatments subsidized by the welfare services [7]. In February 2019, Israel instituted a dental care reform for older adults. According to this reform, people aged 75 and older are eligible for one periodic dental checkup per year and one dental hygienist visit every 6 months, free of charge. Other types of dental treatments to preserve good dentition and prevent future dental disease are available at modest deductible fees ranging from 34 to 136 New Israeli Shekels (about $10–$40) per treatment. A recent study by Sgan-Cohen et al. notes that the current dental component of the Israeli National Health Insurance Law covers children and people aged 75 and over but excludes adults aged 19–74. The authors call for government coverage for all Israelis regardless of age [8].

The current study examines the factors that influence older adults in Israel in deciding whether to undergo routine dental examinations and treatments. To the best of our knowledge, there is a lacuna in the research examining this topic in Israel. The aim of the current study is to fill this void.

## Literature review

Compliance with routine dental examinations and treatments has been linked to various factors, among them socio-demographic, economic, and psychological factors as well as attitudes and beliefs toward oral hygiene and dental treatments. Various studies have examined the factors affecting oral hygiene [9–11]. Some found that older adults with poor physical health and low financial and social status are more likely to have untreated dental issues as well [12–14].

A recent meta-analysis points to major global inequalities in dental service utilization. [12]. Based upon data from the international SHARE survey (Waves 2–3), Listl [15] examined the rate of social-financial inequality in routine dental examinations throughout the life span of people aged 50 and above in 13 European countries. Overall, routine dental care and preventive treatment were found to be comparatively high in five countries (the Netherlands, Sweden, Denmark, Germany and Switzerland) and comparatively low in another five (Spain, Italy, France, Greece, Poland and Ireland). In all the participating countries, a higher level of education was associated with greater chances of undergoing routine dental examinations throughout life. In most of the participating countries, the level of inequality in dental examinations remained constant throughout life.

Zini et al. [16] conducted a study among disadvantaged cohorts of older adults in Israel. The study found that people who are unmarried, live independently at home, are cared for by a family member, were born in Western countries, and have income from a pension are more likely to get routine dental examinations. Zlotnick et al. [17] found that the use of primary dental services increased between 2000 and 2010 in Israel. Yet, this study also found disparities in primary dental care use based on income, immigrant and ethnic minority status, and health risks such as smoking. The analytical model in Soskolne et al. [5] based on data waves 1 and 2 of the SHARE survey for people aged 65 and over in Israel yielded similar results. The model’s findings indicate that age, economic status, and interview language were significant factors affecting the probability of seeking dental treatment. In another study conducted among older adults in Israel, 17% reported that their quality of life was affected by oral hygiene and dental health issues [18]. Those who had visited a dentist within the past year, had their own teeth and no dentures, and reported no chewing problems had better dietary intake [19].

Dental treatments costs are considered a significant obstacle to dental care, pointing to financial inequality [20–22]. For example, Singh et al. [21] showed that poorer oral health among adults in Australia is related to lower levels of household income. Hakeberg and Wide Boman [22] found associations between refraining from dental care for financial reasons and socioeconomic status among adult individuals. These studies clearly point to a need to reduce this inequality by including dental health costs within national health insurance or other health insurance coverage.

McIntyre, Theide, and Birch [23] proposed a framework model for consumption of dental health services. The model included three dimensions that affect willingness to seek treatment: a) affordability, which refers to financial access or ability to afford goods or services; b) availability (physical access); and c) acceptability (cultural access). Yet based on the SHARE survey, Listl [24] found that only a small proportion of older adults (ranging from 7% in Israel to 0.5% in Austria) cited financial reasons for foregoing dental treatments.

In addition to the literature highlighting the importance of socio-demographic factors in the use of preventive health services, including dental examinations, a large body of literature discusses the role of health beliefs and preventive health behavior. This literature is based on theoretical models such as the Health Belief Model (HBM), Reasoned Action, and others that encompass beliefs regarding health and obstacles that prevent healthy behavior. The HBM was developed by Rosenstock et al. to explain why people attend programs for illness prevention or diagnosis [25]. The model assumes that individuals’ behavior is affected by their beliefs regarding the subjective values of the outcomes as well as their subjective expectations regarding the possibility that the preventive behavior will achieve the desirable outcome. The factors affecting preventive health behavior include personal perceived susceptibility to illness (e.g., assessment of risk of dental and oral problems), perceived disease seriousness (e.g., dental problems), perceived benefits of preventive actions (e.g., routine dental examinations), and barriers to taking such actions (e.g., treatment costs, fear of examination). The HBM has been implemented in numerous studies examining health issues in general and dental health issues in particular [26–34]. For example, according to the theoretical study by Flaer et al. [30], the following HBM categories affect preventive dental health behavior: personal perception of probability of dental problems, perceived seriousness of dental illness, sense of self-efficacy, and cues to action (e.g., examination recommendations). According to another recent study, the valid and reliable HBM-based questionnaire can be used to identify the potential barriers to optimal oral health behavior among pregnant women [34].

Most of the aforementioned articles examined the impact of the HBM on the decisions made by individuals from various population groups to take preventive actions to maintain oral hygiene and dental health. Yet the existing literature lacks studies examining the psychological and behavioral factors associated with decisions made by older adults with respect to their oral health. The current study adds to the existing literature through an empirical examination of the HBM principles as well as the socio-demographic and health factors associated with older adults’ decision to undergo routine dental examinations in Israel.

### Hypothesis

Based on the Health Belief Model, we developed the following hypothesis regarding the decision to undergo routine dental checkups: Deciding whether to undergo routine dental checkups will depend on individuals’ attitudes and beliefs with respect to these checkups. In addition, the decision will depend on socio-demographic variables and economic status. More specifically, those who perceive they are more susceptible to dental diseases, believe that dental problems are serious, believe that dental exams have more benefits than drawbacks, have fewer barriers to dental checkups, and have higher levels of health motivation will be more likely to undergo routine dental checkups and will have stronger intentions to undergo checkups the following year.

This hypothesis is based partially on the theoretical HBM prediction in Flaer et al. [30] and on the empirical findings in Shahrabani et al. [32] with respect to mothers’ decisions to take their children for dental examinations.

## Methods

In 2017, a professional polling company conducted a telephone survey among a sample of Israeli adults aged 50–74. The aim of this survey was to examine whether older adults in Israel had undergone dental checkups during the past year, as well as to examine the frequency with which they undergo routine examinations and, more importantly, their intentions to undergo dental checkups in the following year.

### Sample and sampling

The sample the polling company selected for the telephone survey was initially based on probability sampling of groups defined by socio-demographic characteristics.^2^ The sample size was chosen based on the distribution of these sub-groups according to socio-demographic variables and residential area. The sample included 608 respondents: 429 from the Jewish population and 179 from the Arab population. The filter question was: “Is there at least one individual in the household between the ages of 50 and 75?” The 1035 households that were sampled constituted the research population. Of these, 608 returned completed questionnaires (58.74% response rate). Of the remaining questionnaires, 115 did not respond, 301 refused to participate, and 11 answered partially. The interviews were conducted in Hebrew, Russian and Arabic.

### Questionnaire

The questionnaire comprised the following parts:

1. Personal details: socioeconomic information; age; marital status; education; nationality; year of immigration; degree of religiousness (1 = not at all religious, 5 = very religious); household income (1 = above average, 5 = much lower than average); place of residence; health maintenance organization membership; supplemental insurance and/or private dental insurance; accessibility to dental clinics.
2. Frequency of regular dental checkups and whether participant had dental checkups during the last 2 years. Intention to undergo a dental checkup during the next year (1 = certainly yes, 5 = certainly no). Did participant undergo dental treatments after problems were diagnosed by routine tests? Did participant forego dental treatments because of financial cost? Did participant receive recommendation to get dental checkups? To what extent is participant concerned about dental treatments (1 = not at all, 5 = very much)? Does participant believe that “you only need to visit a dentist when you are in pain”?
3. Perceived health status and oral health status (1 = very good, 5 = very bad); number of missing teeth; extent to which participant maintains routine oral hygiene.
4. HBM variables by categories: perceived importance of dental checkups; perceived seriousness of dental problems; perceived susceptibility to dental problems; perceived benefits of checkups; barriers to checkups; and health motivation.^3^

The model’s constructs are illustrated in Table 4 (Appendix). Response possibilities ranged from 1 = strongly agree to 5 = strongly disagree. Additionally, the questionnaire included questions concerning degree of trust in the dentist and extent of fear of dental checkups. The questionnaire was translated into Hebrew, Arabic and Russian. In the first stage, a pilot questionnaire was administered to 50 individuals, and after improvements were made, the final format was developed.

### Statistical data analysis methods

SPSS 20 software was used for statistical analysis of the data. Chi-square testing was used to define the relationship between the categorical variables (including personal factors) and the dependent variables: frequency of checkups and intention to get a checkup in the following year. One-way ANOVA was used to determine the statistical significance of the differences in the averages of the sequential variables (e.g., scales of measure for HBM variables) among the different groups. Cronbach alpha was calculated for the HBM constructs. Additionally, two types of regression analysis were used: a) ordered logistic regression to identify the influence of the demographic variables, the HBM categories, and additional variables concerning the use of dental services, and b) logistic regression to identify the intention to get a checkup the following year.

## Results

### Use of dental services according to socio-demographic and other variables

The total sample included 608 participants: 47.2% reported getting dental checkups at least once a year, 7.5% once every 2 years, and 45.3% less often than every 2 years. In addition, 75.4% indicated a strong intention to undergo checkups in the next 12 months, compared to 18.9% of the sample that indicated weak or no intentions of getting dental checkups in the next 12 months and 5.7% who were undecided about whether to undergo checkups in the next year.

Table 1 summarizes the distribution of the entire sample according to different characteristics (column 3). Additionally, Table 1 compares the percentage of each characteristic according to: a) frequency of dental checkups (columns 4 and 5 compare the percentages of those who get dental checkups every year with those who get checkups less frequently than every 2 years, if at all)^4^; b) intention to undergo checkups in the next 12 months (columns 7 and 8 compare the percentages of those who intend to get checkups with those who do not).^5^

**Table 1:** Survey data—Frequency of dental checkups and intention to undergo dental examination according to socio-demographic and other characteristics

	Entire sample (*N* = 608)	Frequency of dental checkups			Intention to undergo dental checkups in the next 12 months		
Variable	%	Once a year (%)	Less than two years (%)	Chi square (*p* value)	Intend (%)	Do not intend (%)	Chi square (p value)
Gender							
Women	51.3	56.5	43.5	2.66 (.103)	84.7	15.3	8.25 (.004)^***^
Men	48.7	49.4	50.6		75.0	25.0	
Age							
50–59	46.9	57.9	42.1	5.99 (.050)^**^	84.1	15.9	14.61 (.001)^***^
60–69	37.1	50.7	49.3		80.6	19.4	
70+	16.0	43.0	57.0		65.6	34.4	
Religion							
Jewish	69.4	55.6	44.4	4.10 (.043)^**^	80.4	19.6	0.20 (.657)
Other	30.6	46.2	53.8		78.7	21.3	
Marital status							
Married	82.7	52.5	47.5	0.15 (.702)	78.9	21.1	1.47 (.226)
Not married	17.3	54.6	45.4		84.4	15.6	
Family income							
Average and above	53.4	59.5	40.5	14.17 (.000)^***^	83.0	17.0	4.19 (.041)^**^
Below average	46.6	42.3	57.7		75.6	24.4	
Education							
Up to 12 years	16.6	38.6	61.4	8.68 (.003)^***^	67.7	32.3	10.68 (.001)^***^
More than 12 years	83.4	55.8	44.2		82.4	17.6	
Private dental insurance							
Yes	18.2	65.6	34.4	7.81 (.005)^***^	89.0	11.0	6.57 (.010)^***^
No	81.8	49.7	50.3		77.6	22.4	
Oral health status							
Good and above	78.5	57.8	42.2	16.94 (.000)^***^	80.6	19.4	0.52 (.473)
Bad	21.5	36.2	63.8		77.7	22.3	
Oral hygiene							
Low or average	15.2	34.1	65.9	(^***^13.92.000)	69.8	30.2	6.86 (.009)^***^
Above average	84.8	56.5	43.5		82.0	18.0	
Dental treatment recommendation							
Yes	23.1	56.1	43.9	0.66 (.418)	85.9	14.1	4.19 (.041)^**^
No	76.9	51.9	48.1		77.8	22.2	

^***^ *p* < 0.01, ^**^ *p* < 0.05, ^*^ *p* < 0.1

Table 1 shows that the rate of annual dental checkups is significantly higher among Jews, younger participants, those with higher education, those with higher household income, those with dental insurance, those who assess their oral health status as good, and those who maintain good oral hygiene.

Table 1 also shows that the rate of intention to undergo dental checkups in the next 12 months is significantly higher among women, younger participants, those with higher education, those with higher household income, those with dental insurance, those who maintain good oral hygiene, and those who received a recommendation to undergo dental checkups.

Additionally, participants in the sample who were asked why they avoid routine dental checkups gave the following main reasons: a) financial cost (45.9%), b) lack of time (30.8%), c) fear of pain (23.8%).

### Results for HBM categories and attitudes

Table 2 shows the mean values and standard deviations for the HBM model categories and for additional variables, including degree of trust in the dentist, dentist availability, fear of dental checkups, degree of avoiding dental care due to high financial costs, and degree of agreement with the statement: “you only need to go to the dentist when you’re in pain.” The mean values of these variables are shown according to frequency of dental checkups and intention to undergo routine dental checkups in the next 12 months. The Cronbach’s alpha coefficients are reported in Table 4 in the Appendix.^6^

**Table 2:** Mean and Standard Deviations of HBM variables and attitudes by frequency of checkups and by intentions to undergo checkups next year

	Dental checkup frequency			Intention to undergo dental checkups next year		
	Once a year *N* = 285	Less than 2 years *N* = 253	F (*p* value)	Intend *N* = 453	Do not Intend *N* = 114	F (*p* value)
	Mean(SD)			Mean(SD)		
Perceived importance^a^	4.76 (0.39)	4.61 (0.52)	14.35^***^ (.000)	4.75 (0.38)	4.46 (0.63)	37.09^***^ (.000)
Perceived seriousness^a^	4.83 (0.44)	4.71 (0.66)	6.79^***^ (.009)	4.81 (0.52)	4.63 (0.71)	9.14^***^ (.003)
Perceived benefit^a^	4.76 (0.40)	4.58 (0.59)	17.57^***^ (.000)	4.74 (0.40)	4.44 (0.71)	34.57^***^ (.000)
Barriers^a^						
Dental treatments are time consuming	3.73 (1.34)	3.91 (1.25)	2.46 (.118)	3.79 (1.30)	3.90 (1.33)	0.56 (.453)
Dental treatments are expensive	4.74 (0.68)	4.76 (0.64)	0.09 (.762)	4.76 (0.65)	4.74 (0.66)	0.15 (.697)
Dental treatments are too risky	2.91 (1.43)	3.08 (1.49)	1.62 (.204)	2.98 (1.45)	2.98 (1.52)	0.00 (.978)
Susceptibility^a^	2.86 (1.34)	2.91 (1.5)	0.14 (.707)	3.01 (1.39)	2.70 (1.51)	3.95^**^ (.047)
Dentist availability^a^	4.56 (0.92)	4.18 (1.25)	17.03^***^ (.000)	4.45 (1.02)	4.16 (1.32)	6.21^**^ (.013)
Trust in dentist^a^	4.51 (0.77)	4.24 (0.93)	12.95^***^ (.000)	4.43 (0.80)	4.23 (1.06)	4.26^**^ (.039)
Fear of dental treatments^b^	1.93 (1.18)	2.13 (1.35)	3.47^*^ (.063)	2.05 (1.25)	1.93 (1.30)	0.78 (.377)
Health motivation^a^	4.27 (0.92)	3.69 (1.23)	39.19^***^ (.000)	4.12 (1.04)	3.64 (1.24)	18.26^***^ (.000)
Avoiding dental treatments due to high costs^b^	1.96 (1.48)	2.74 (1.71)	31.90^***^ (.000)	2.26 (1.62)	2.55 (1.71)	2.89^*^ (.090)
You only need to go to the dentist when you’re in pain.^a^	2.01 (1.52)	2.61 (1.62)	19.69^***^ (.000)	2.11 (1.51)	2.94 (1.68)	25.73^***^ (.000)

^***^ *p* < 0.01, ^**^ *p* < 0.05, ^*^ *p* < 0.1

^a^ 5-level scale ranging from (1) “not agree at all” to (5) “very much agree”

^b^ 5-level scale ranging from (1) “very little or not at all” to (5) “very much”

The results in Table 2 suggest that, in line with our hypothesis, frequency of dental checkups and intention to undergo a checkup in the next 12 months were significantly *higher* for those who: a) perceived dental health to be more important; b) perceived dental problems to be more serious; c) perceived the benefits of dental checkups to be greater; d) perceived that dentists were more available; e) were less likely to forego dental treatments because of financial costs; f) believed to a lower extent that "you only need to go to the dentist when you’re in pain; and g) have higher health motivation. In addition, the frequency of dental checkups was significantly higher among those who have a higher degree of trust in dentists, while the intention to undergo checkups was significantly higher among those who perceived they were more susceptible to dental problems.

### Results of analytical model

Table 3 summarizes the results of the two regressions analyses. The first analysis (Table 3, columns 2–3) shows the results of the ordered logistics regression that examined the factors influencing the dependent variable: frequency of dental checkups (coded as five categories: once a year, once every 2 years, once every 3 years, less than once every 3 years, and not at all). The second analysis (Table 3, columns 4–5) shows the results of the logistics regression that examined the factors influencing the dependent variable: intention to undergo a dental checkup the following year (0 = do not intend to undergo, 1 = intend to undergo; the category “do not know” was omitted).

**Table 3:** Results of the analytical survey model: Factors influencing checkup frequency and intention to undergo dental checkups

	Frequency of dental checkups^a^		Intention to undergo dental checkups (base = no)	
Explanatory variables	Beta coefficient	Std. Err	Beta coefficient	Std. Err
Gender (base = men)	0.151	0.180	0.363	0.254
Age (base = 50–59)	−0.547^**^	0.257	−0.556^**^	0.167
Marital status (base = married)	0.236	0.242	0.892^**^	0.389
Household income (base = average and above)	−0.511^***^	0.196	−0.496^*^	0.274
Nationality (base = Jewish)	−0.678^***^	0.209	−0.307	0.287
Perceived benefits	−0.536^**^	0.222	0.745^***^	0.281
Perceived importance	−0.116	0.238	0.650^**^	0.292
Perceived dentist’s availability	−0.263^***^	0.082	0.173^*^	0.105
Health motivation	−0.421^***^	0.084	0.383^***^	0.110
Recommendation (base = yes)	−0.262	0.212	−0.516^*^	0.311
Constant			−5.800^***^	1.581
	*N* = 510, Chi-square = 80.098, *p* < 0.000		*N* = 494, Chi-square = 66.611, *p* < 0.000	

^*^p < 0.1, ^**^p < 0.05, ^***^p < 0.01

^a^The scale was: 1 = once a year, 2 = once every 2 years, 3 = once every 3 years, 4 = less than once every 3 years, 5 = not at all

The explanatory variables for the regressions were as follows: age group, gender, family income, nationality, marital status, whether participant received recommendation to undergo a dental checkup, and the HBM categories of perceived benefits, perceived importance of dental checkups, dentist availability, and health motivation.^7^

The results in Table 3 (columns 2–3) show that after controlling for the rest of the explanatory variables, the variables that significantly influenced frequency of dental checkups were: a) the HBM categories of perceived benefits of dental examinations and health motivation; more specifically, the chances of getting frequent checkups rise as the perceived benefits of dental checkups and the level of health motivation increase; b) perceived availability of dentists; the chances of getting frequent checkups increase as the perceived availability of dentists increases; c) socio-demographic characteristics: lower age, higher income, and being Jewish predicted higher chances of getting frequent checkups. These findings are compatible with our hypothesis.

The results in Table 3 (columns 4–5) show that the following variables significantly influenced the intention to undergo a dental checkup the following year. First, the three HBM categories—perceived benefits of dental examinations, perceived importance of dental examinations, and health motivation—exerted a major impact. In addition, recommendation for checkups and dentist availability exhibited a marginal effect. More specifically, the intention to get a checkup in the following year increases as the following factors increase: individuals’ perceived benefits and perceived importance of dental checkups, perceived availability of dentists, level of health motivation, and recommendations for dental checkups. These findings are compatible with our hypothesis. Second, the following socio-demographic characteristics also predicted greater intentions of getting dental checkups: lower age, being single, and having higher income levels.

## Discussion

The results of a telephone survey among a national sample of Israelis aged 50–75 suggest that fewer than 50 % (47.2%) get dental checkups at least once a year, while most (52.8%) undergo dental checkups less frequently, if at all. Nevertheless, most of the participants (75.4%) indicated strong intentions of undergoing checkups in the next 12 months.

The current study empirically confirms the theoretical prediction of the HBM model [31] with respect to dental checkups among older people. The findings indicate that the chances of getting frequent checkups increase as the following factors increase: perceived benefits of dental checkups, perception of dentist availability, and level of health motivation. In addition, the findings indicate that the intention to get checkups in the following year increases as the following factors increase: individuals’ perceived benefits and perceived importance of dental checkups, perceived dentist availability, level of health motivation, and recommendation to get dental checkups.

These results with respect to the HBM categories are compatible with the findings of Shahrabani et al. [32] that dental checkups were significantly more frequent among children whose mothers perceived dental health as more important, perceived routine dental checkups as more beneficial, and had fewer barriers to dental checkups. Yet, as predicted by the HBM theoretical study of Flaer et al. [30], the current study did not find support for other HBM categories, such as perceived probability of dental illness and perceived seriousness of dental illness. One possible explanation is that the effect of health beliefs on health prevention behavior and intentions depends on the study population. The psychological factors affecting young people may be different than those affecting people aged 50 and above.

The results of our analytical model also show that the use of dental services and the intention to undergo dental checkups are significantly greater among socioeconomically strong populations. Lower age, higher income levels, and being Jewish predicted greater chances of getting frequent checkups, while lower age, being single, and higher income levels predicted greater intentions to get dental checkups. These results emphasize the unequal use of dental services among older people in Israel in that those with high economic status tend to make more use of dental services than those with low economic status. These results are also compatible with previous studies that found that older adults with low financial and social status are more likely to have untreated dental issues [12, 13].

The current study has some limitations. One is that the questionnaire included recall behavior questions regarding participants’ dental checkups. Recalled answers can be inaccurate and can also be affected by social desirability bias. Yet, the majority of the participants (52.8%) reported that they had not undergone dental checkups during the last year, while most (75.4%) declared they intended to get dental checkups in the coming year.

## Conclusions

Under the recent Israeli dental care reform for the older population, people aged 75 and over are eligible for dental checkups free of charge and basic treatments at a modest deductible fee. The results of the current research with respect to people aged 50–75 underscore the need to implement this dental reform in Israel for those younger than 75, since the majority of those in this age group do not get the necessary dental checkups. Expanding the dental reform to older adults under 75 can minimize gaps in Israeli society regarding dental health for older adults.

In view of the finding that perceived availability of dentists is a significant factor in the probability of getting dental treatment, broader national distribution of dental clinics is required to provide older adults suitable access to dental care. Moreover, health services for the older adult population should be planned according to beliefs and values acceptable among this population group [35].

The findings of the current study indicate that those who speak Arabic are less likely to undergo dental treatments than those who speak Hebrew. Therefore, in order for medical treatment to be effective, it must take into consideration the cultural aspects of the target population (e.g., language). This conclusion is in line with the findings of Marino et al. [36] with respect to other countries.

Another conclusion emerges from the findings that health beliefs affect the probability and intention to undergo routine dental examinations: This population group should be the target of systematic health education through the media and health maintenance organizations. The information should emphasize the importance of dental health and the benefits of routine checkups in preventing dental problems, thus encouraging older adults to get dental checkups more frequently. In fact, systematic health education with respect to oral health may also help in successfully implementing the new dental reform for people aged 75 and over.

## Appendix

**Table 4:** Health beliefs model (HBM) categories and other variables^*^

	Category	Cronbach Alpha	Questionnaire items
1	Perceived importance of dental health	0.651	• Dental health is very important
			• Routine dental examinations are very important
			• Treating dental issues is as important as treating other health issues
			• Routine dental examinations prevent dental and gums issues
			• Routine dental hygienist treatments prevent dental and gums issues
2	Perceived benefits of dental examinations	0.676	• Routine dental examinations will prevent dental and gums issues
			• It is important to take care of your teeth in order to save money that can later be used for treating severe dental issues
			• Dental treatments and examinations help maintain teeth for a long time
3	Perceived barriers to dental examinations		• Dental treatments and examinations are time consuming procedures
			• Dental treatments costs a lot of money
			• Dental treatments are too risky
4	Perceived susceptibility	0.810	• There is a high probability that in the future you will have dental and gum issues
			• In your opinion, during the coming year you will suffer from dental or gum issues
5	Perceived seriousness		• Dental and gum diseases may cause teeth loss
6	Dentist availability		• When I have dental issue, dentists are available
7	Trust in dentist		• How much trust do you have in your dentist?
8	Fear of dental examinations	0.903	• Concerned
			• Afraid
			• Terrified
			• Nervous
9	Health motivation	0.670	• You get periodic examination every year, in addition to visiting the doctor when you are ill
			• You usually act according to medical recommendations because you believe they will improve your health

*Scale levels ranging from (1) “do not agree at all” to (5) “very much agree”

## Abbreviations

HBM: The Health Belief Model
SHARE: Survey of Health, Aging and Retirement in Europe
WHO: World Health Organization

## Footnotes

1 In 2012, there were 69.1 dentists for every 10,000 persons in the Israeli population [37].

2 The survey was conducted by a professional institution for public opinion surveys (Cohen Institution, affiliated with Tel Aviv University).

3 This part of the questionnaire was based partially on the HBM questionnaires developed by Flaer et al. [30], Nakazono et al. [31] and Buglar et al. [38].

4 The category “every 2 years” was omitted to emphasize the difference between the two other categories.

5 The category “do not know” was omitted for the same reasons.

6 The Cronbach’s alpha coefficients for all categories were above 0.65, except for the category of perceived barriers, which was lower. Therefore Table 2 displays separate constructs for the barriers.

7 In several versions of the regression analyses we used various HBM categories and other control variables, including health status and dental status variables. The final versions of the regression analysis are presented after the regression analysis was fine-tuned and the steps with insignificant variables were removed.
